# Loss of kidney function due to proteinuria, common problem with a rare cause: Question

**DOI:** 10.1007/s00467-020-04521-7

**Published:** 2020-03-11

**Authors:** Julia Steinke, Michaela Gessner, Leonie Frauenfeld, Anna K Fischer, Wiebke Solass

**Affiliations:** 1 Institute of Pathology and Neuropathology, University Hospital Tuebingen, Eberhard-Karls University Tuebingen, Tuebingen, Germany; 2 Department of Pediatric Nephrology, University Hospital Tuebingen, Eberhard-Karls University Tuebingen, Tuebingen, Germany

## Case study

We report a 12-year-old girl with a large unselective proteinuria since she was 7 years old. To delay the progression of proteinuria/renal impairment, medication with ramipril and candesartan was introduced and maintained for several years. Secondary complications of renal insufficiency, such as renal anemia, secondary hyperparathyroidism, and metabolic acidosis, were treated conservatively.

In addition, the patient had significant growth retardation (− 3.53 SDS), myopia, and a mild aortic valve insufficiency. The developmental neurological examination was unremarkable.

**Fig. 1:**
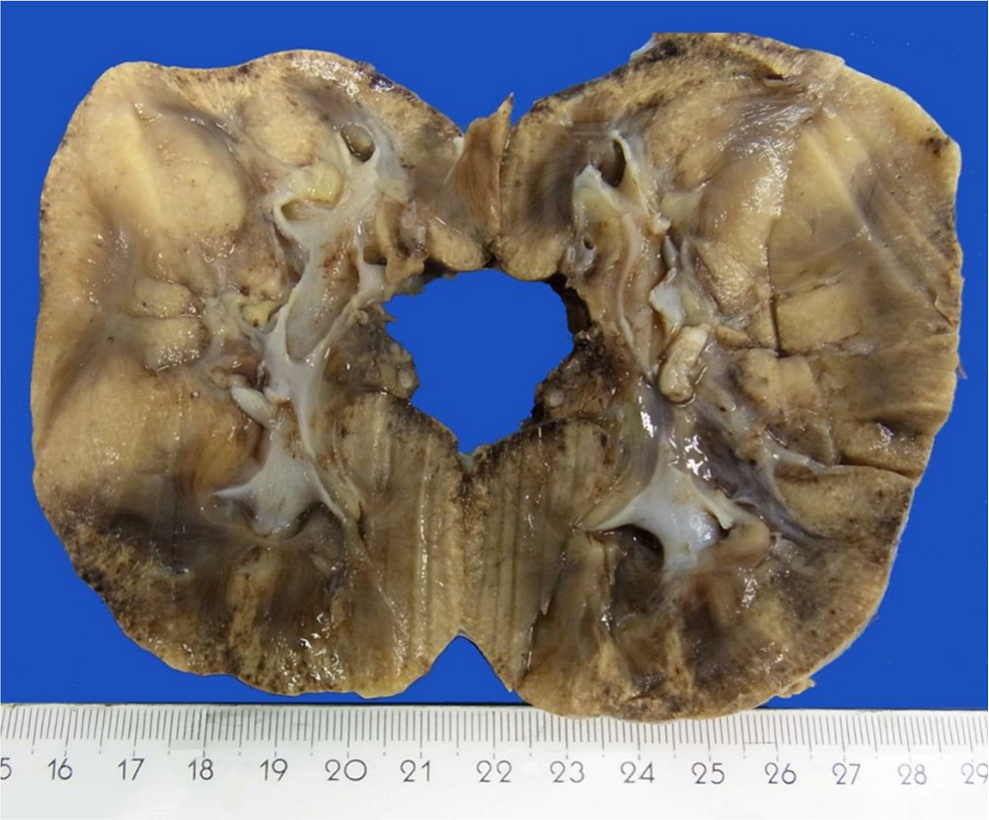
Nephrectomy-specimen tangential sectioned; note the color changes between medulla and cortex; the lower and upper half of the kidney. Especially on the left upper half a pronounced yellowish discoloration of the tissue is noticed

**Fig. 2:**
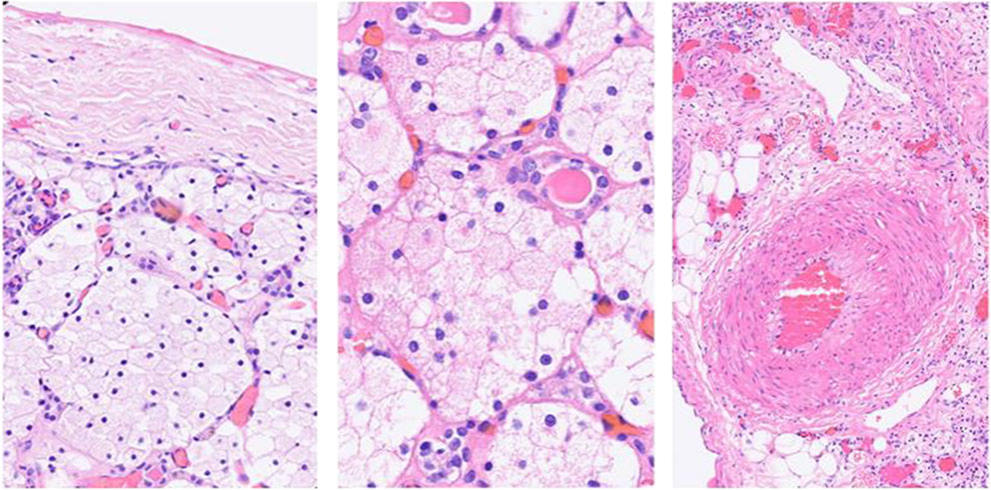
Histological examination of the nephrectomy. Renal parenchyma with massive, interstitial accumulation of histiocytic macrophages with clear, fine coarse cytoplasm (middle). The foamy macrophages are preferentially located at the periphery of the organ (left). The renal vessels show regular architecture (right)

However, when she was 12 years old, the consequences of proteinuria became unmanageable; she presented increasing edema and ascites (laboratory tests shown in Table 1). Attempts to stabilize the situation with albumin substitution and furosemide failed. She developed renal failure and a hemodialysis catheter was implanted. In the same session a nephrectomy of the left kidney was performed to minimize the loss of albumin. The day after nephrectomy, the proteinuria had halved (see image of nephrectomy specimen in Fig. 1 and histological results in Fig. 2). However, a few days after the initial operation she presented oligo-/anuresis. The remaining right kidney could not provide sufficient diuresis. Therefore, hemodialysis was started, maintained for 2 years (three times a week) before kidney transplantation was successfully performed. The then performed right-sided nephrectomy specimen showed the same macroscopic and histological changes as its counterpart.

**Table 1:** Laboratory results at hospital admission

	Value	Range	Unit
Laboratory blood test			
Erythrocyte	3.38	4.0–5.2	Mio/μl
Hematocrit	24.8	37.0–47.0	%
HB	8.8	11.8–15.0	g/dl
MCH	26	27.0–34.0	pg
MCHC	35.5	32.0–36.0	g/dl
MCV	73.4	80–93	fl
Sodium	143	136–148	mmol/l
Potassium	4.4	3.4–4.8	mmol/l
Calcium	2.1	2.1–2.6	mmol/l
Phosphate anorganic	2.0	1.3–1.8	mmol/l
Creatinine	3.8	0.2–0.6	mg/dl
Urea	180	10–35	mg/dl
Cystatin C	3.4	0.5–1.0	mg/l
Protein total	5.1	6–8	g/dl
Albumin	2.7	3.0–3.0	g/dl
Cholesterin	160	130–190	mg/dl
Triglyceride	213	max. 200	mg/dl
C-reactive protein	0.04	max. 0.05	mg/dl
PTH	35.7	1.5–7.6	pmol/l
Laboratory urine test			
Crea	52		mg/dl
Protein	13.1	max. 0.1	g/l
Quot. U-Protein/U-Crea	25,192	max. 100	mg/gCrea
Albumin	13,000	max. 20	mg/l
Quot. U-Alb./U-Crea	25,000	max. 20	mg/gCrea
A1-Microglob./Crea	148.1	max. 13	mg/gCrea
U-IgG/gCrea	1271.2	max. 10	mg/gCrea

## Question

What is the cause of the nephrotic syndrome in this patient?

